# Effects of short-chain fatty acid-producing probiotic metabolites on symptom relief and intestinal barrier function in patients with irritable bowel syndrome: a double-blind, randomized controlled trial

**DOI:** 10.3389/fcimb.2025.1616066

**Published:** 2025-06-12

**Authors:** Erfeng Li, Jie Wang, Bin Guo, Wenbin Zhang

**Affiliations:** Department: Endoscopy Center, Shanxi Province Cancer Hospital/Shanxi Hospital Affiliated to Cancer Hospital, Chinese Academy of Medical Sciences/Cancer Hospital Affiliated to Shanxi Medical University, Taiyuan, Shanxi Province, China

**Keywords:** Irritable bowel syndrome (IBS), Probiotics, Short-chain fatty acids (SCFA), Intestinal barrier function (IBF), double-blind randomized controlled trial (DBRCT)

## Abstract

**Background:**

IBS often appears as bloating, altered bowel patterns, and abdominal pain (AP).Probiotics and SCFA may be useful in mucosal repair and symptom relief, according to earlier research, however there is currently a lack of systematic evidence supporting their therapeutic effectiveness across a variety of IBS subtypes.

**Objective:**

To investigate the impacts of probiotics on signs and intestinal barrier function (IBF) in individuals with multiple IBS subtypes, and evaluate the role of SCFA in this process.

**Methods:**

A double-blind randomized controlled trial (DBRCT) design was adopted. Using the Rome IV criteria, a total of 120 individuals with IBS were randomised to either the probiotic group (PG) or placebo group (PLG). The intervention lasted for 12 weeks with an additional 4-week follow-up. In addition to fecal SCFA (FSCFA) levels, intestinal permeability (L/M ratio), tight junction proteins (TJP), serum/fecal inflammatory markers, and adverse event occurrence, the primary endpoint (PEP) evaluated was IBS Symptom Severity Scale (IBS-SSS) scores. Subgroup analysis was performed in selected cases.

**Results:**

In terms of symptom scores, there was a major correlation among group and time (F=9.314, P<0.001), and repeated-measures ANOVA showed that the PG’s scores were considerably < than those of the control group (CG) beginning in week 8 (all P<0.01). Levels of acetate, propionate, and butyrate considerably increased after 12 weeks of intervention (all P<0.01). Intestinal permeability and Occludin significantly improved at weeks 8 and 12 (all P<0.0167), while important differences in Claudin-1 and Zonulin appeared only at week 12 (all P<0.0167). Inflammatory markers considerably decreased at week 12 (all P<0.0167). There were no statistically significant differences in adherence or adverse events (P>0.05). Reductions in symptom scores were positively connected with an increase in SCFAs (r=0.43, P=0.002). Subgroup analysis across multiple IBS subtypes indicated significant symptom relief at week 12 for all subtypes (all P<0.05).

**Conclusion:**

Probiotics significantly improved clinical symptoms in IBS patients of different subtypes by increasing short-chain fatty acid levels, repairing the intestinal barrier, and reducing inflammation.

## 1 Introduction

Abdominal pain, bloating, and defecation difficulties are symptoms of IBS, a prevalent functional gastrointestinal condition in clinical practice that significantly impairs social functioning and standard of living for a significant percentage of the global population (Yu et al., 2025; Lei et al., 2025; Lovell and Ford, 2012). Although its exact etiology is not yet entirely comprehensible, dysbiosis of the gut microbiota (GM), mucosal barrier defects, and chronic low-grade inflammation are regarded as possible common mechanisms. There is currently insufficient clinical evidence to determine whether probiotics can be widely applied to various subtypes of IBS and whether they can consistently and sustainably improve symptoms, despite existing studies highlighting their critical role in balancing the intestinal microbial ecosystem and reducing inflammatory responses.

(Gralnek et al., 2000; Yang et al., 2024; Gasiorowska et al., 2024; O’Mahony et al., 2005; Mazurak et al., 2015). Many explorations have focused primarily on short-term interventions or observation of individual inflammatory markers, without a multidimensional, systematic evaluation of symptom changes, mucosal barrier repair, and inflammation down-regulation (Azarfarin et al., 2024; Ford et al., 2018). The lack of research involving multiple subtypes, multiple indicators, and extended follow-ups makes it difficult to provide precise protocols tailored to different patient characteristics in clinical practice (Almabruk et al., 2024; So et al., 2023). The theoretical basis for the interventional advantages of SCFA includes providing energy to the intestinal epithelium, inhibiting intestinal inflammation, and protecting barrier function, but many key aspects remain insufficiently clarified, especially regarding the assessment of their combined effect with probiotics on the overall efficacy in multiple types of IBS patients, which still requires further validation (Lopes et al., 2024; Hamer et al., 2008). Through a rigorous DBRCT under standardized baseline intervention conditions, individuals with different subtypes of IBS, this study methodically examines the clinical symptom relief and possible mechanisms of probiotics. By comprehensively evaluating symptom scores, short-chain fatty acid levels, intestinal barrier permeability, and changes in inflammatory mediators, this study elucidates the “probiotics—short-chain fatty acids—intestinal barrier—clinical symptoms” pathway. The longer intervention period and multidimensional index analysis in this study provide a reliable basis for individualized clinical interventions and lay the groundwork for a broader implementation of microecological therapy. The study results can offer direct and robust evidence-based support for optimizing probiotic formulations and combined dietary strategies.

## 2 Materials and approaches

### 2.1 Study subjects

Inclusion criteria: ① Age 18–65 years, no restriction on gender; ② The Rome IV criteria (Ford, 2024) for diagnosing IBS include a minimum of six months of symptoms and the exclusion of any organic intestinal lesions; ③ No use of antibiotics, systemic probiotic preparations, or other medications affecting the gut microbiota in the past 2 months; ④ No severe cardiovascular, hepatic, renal, metabolic, or neuropsychiatric diseases in the decompensated stage; ⑤ Able to comply with and complete the research requirements, and voluntarily sign an informed consent form.

Exclusion criteria: ① Verified ulcerative colitis, colorectal tumors, or Crohn’s disease; ② Pregnant or lactating women; ③ Major surgery within the last 3 months or acute infectious diseases; ④ Participation in other interventional clinical trials within the last month, or lack of good compliance; ⑤ Allergy to the investigational preparation or the placebo components.

The Medical Ethics Committee of XX Hospital gave its approval to this study protocol (Approval No.: XXXX). All participants voluntarily signed a written informed consent form before to the study’s commencement after being fully informed about its objectives and any risks.

### 2.2 Study design and grouping

The DBRCT study conducted at this hospital from January 2023 to December 2024. In order to assess the effect of probiotics on IBS symptoms and intestinal barrier function under standardised baseline intervention circumstances, all individuals underwent a 12-week continuous intervention, which was followed by a 4-week follow-up. Following screening and signing the informed consent form, subjects were randomly assigned to either the PG or the PLG in a 1:1 ratio using a computer-generated random number table.

They were then stratified by IBS subtype (diarrhea-predominant, constipation-predominant, mixed, and unclassified). An independent data manager who was not involved in the intervention or testing maintained the random sequence.

The probiotic preparation and placebo were identical in appearance, packaging, and taste. All subjects and clinical research personnel involved in observation remained blinded until the conclusion of the study and completion of data locking.

Preliminary research indicated that the IBS-SSS (IBS-Severity Scoring System) score could decrease by approximately 50 points after probiotic intervention, while the control could decrease by about 20 points, with an estimated standard deviation of 40 points. Each group eventually had 60 subjects, for a total of 120 participants, with α=0.05 and power (1−β)=0.80.

### 2.3 Intervention methods

#### 2.3.1 Unified basic intervention

All subjects followed unified basic intervention measures during the trial to reduce confounding effects related to diet, exercise, and psychological factors, and to approximate real clinical settings. The research nurse gave dietary advice that was low in fermentable oligo-, di-, mono-saccharides and polyols, or FODMAPs. Subjects avoided consuming onions, garlic, and high-fructose syrup, ate meals in divided portions each day, and recorded their food intake, ensuring an appropriate amount of fruits and vegetables. The research personnel assessed compliance during follow-up (Matsuura et al., 2024). Subjects performed at least three sessions of moderate-intensity aerobic exercise (such as running or brisk walking) per week, each lasting at least thirty minutes, and they recorded their daily routine in relation to rest and exercise.

They ensured 7–8 hours of sleep each night, avoided staying up late or excessive overtime, and contacted the research staff for relaxation training if they experienced sleep problems (Khalaidzheva et al., 2024). In terms of psychological and symptomatic management, if subjects showed signs of anxiety or depression, they underwent brief relaxation training and consulted a mental health specialist if necessary. If they experienced evident abdominal pain, diarrhea, or constipation, they could, after standardized documentation, use antispasmodics, antidiarrheal agents, or bulk-forming laxatives, and report medication dosage and duration at follow-up. Both groups received exactly the same basic intervention measures.

#### 2.3.2 Probiotic group

Chr. Hansen A/S in Denmark created the probiotic product in a single batch, and each sachet included a minimum of 1×1010 CFU of a mixture of Lactobacillus and Bifidobacterium strains. Third-party testing confirmed that it met the hygiene and quality standards of the national drug regulatory authority. Subjects took one sachet each morning and one at bedtime, dissolved in approximately 200 mL of warm water, avoiding hot water to protect the viability of the live bacteria, for 12 consecutive weeks, in conjunction with the basic intervention measures. Subjects recorded the time and quantity of each dose, and the research personnel checked the remaining sachets at each follow-up. If severe abdominal pain or a rash occurred during the trial, the subject was required to immediately report to the research team and discontinue use of the preparation, with subsequent safety evaluations carried out by a designated individual.

#### 2.3.3 Placebo group

The placebo was produced in the same batch by the same manufacturer, with each sachet containing starch and a small amount of dextrin, identical in appearance and taste to the probiotic preparation. Subjects took one sachet each morning and one at bedtime, dissolved in 200 mL of warm water, for 12 consecutive weeks, while following the same dietary, exercise, and rest guidelines as the probiotic group. During administration, subjects recorded the number of empty sachets and usage. At each follow-up, the research personnel verified these records and reviewed adverse events. If any serious drug-related adverse event occurred, it had to be reported promptly and handled according to a unified emergency protocol.

### 2.4 Observation indicators and detection methods

At baseline (W0), week four of the intervention (W4), week eight of the intervention (W8), week twelve of the intervention (W12), and week four following the conclusion of the intervention (W16), all indicators were gathered and assessed. Every time point’s testing was carried out at the same time point on the assigned day.

#### 2.4.1 IBS symptom severity scoring

At W0, W4, W8, W12, and W16, the research assistant conducted the IBS-SSS (questionnaire (Dimzas et al., 2024) through a face-to-face approach to guide the subjects. The severity, frequency, degree of bloating, satisfaction with bowel habits, and hindrance with everyday life are the five components of this scale. A total of 0 to 500 points can be earned by scoring each component from 0 to 100.

Severe IBS symptoms are indicated by higher scores. During each visit, the research assistant recorded the total questionnaire score in the electronic data system.

#### 2.4.2 Fecal short-chain fatty acids measurement

At W0, W4, W8, W12, and W16, subjects collected approximately 5 g of fresh feces during their morning bowel movement, immediately placed it in a sterile sampling tube with a sealed cap, and transported it to the laboratory within 1 hour for storage at −80°C. The amounts of acetate, propionate, and butyrate in feces are measured using a GC-MS (Gas Chromatography-Mass Spectrometry) instrument.

GC-MS allowed for the calculation of each compound’s percentage of total SCFAs.The specific procedure included sample pretreatment, derivatization, and quantitative analysis, with results expressed in µmol/g of feces.

#### 2.4.3 Intestinal barrier function testing

At W0, W8, and W12, intestinal permeability (L/M test) was measured. After fasting for a certain amount of time, the subjects drank 50 mL of a solution that contained 10 g of lactulose and 5 g of mannitol.They collected all urine over the following 6 hours, thoroughly mixed it, and set aside 5 mL. The L/M ratio was computed after the quantities of mannitol and lactulose in the urine were measured using high-performance liquid chromatography (HPLC).

At the same times, TJP measurements were made. After 4 mL of fasting venous blood was extracted in the morning and centrifuged for 10 minutes at 3000 rpm, the serum was separated and stored at -80°C.

The serum concentrations of the proteins occludin, claudin-1, and zonulin, expressed in ng/mL, were measured using the enzyme-linked immunosorbent assay (ELISA).Higher values indicate higher tight junction protein abundance and better intestinal barrier integrity.

#### 2.4.4 Inflammatory and immune indicators

Serum was extracted from 4 mL of fasting venous blood at W0, W8, and W12 in the morning and centrifuged for 10 minutes at 3000 rpm. Fecal samples were collected in the same manner as for SCFAs but were aliquoted into separate sterile tubes for testing fecal inflammatory markers. A fully automated chemiluminescence immunoassay analyser was used to quantitatively quantify serum C-reactive protein (CRP), interleukin-6 (IL-6), and tumour necrosis factor-α (TNF-α). The results were expressed in pg/mL.

Fecal calprotectin was quantitatively measured by ELISA, with results expressed in µg/g of stool.

#### 2.4.5 Adverse events and compliance recording

The research assistant collected any subjective discomfort or objective symptoms related to the use of probiotics or placebo, including severe abdominal pain, rash, or other adverse reactions. If non-gastrointestinal symptoms occurred, their duration and any interventions were recorded. The research assistant verified the number of empty sachets turned in by participants and checked daily medication logs to calculate actual adherence; if it fell below 80%, the participant was included in the intention-to-treat (ITT) analysis with a corresponding note.

### 2.5 Quality control

Research assistants and technicians underwent standardized training to become familiar with the processes and protocols for questionnaire scoring, specimen collection, and laboratory testing. All biological samples were labeled after collection and promptly stored in a −80°C freezer. Prior to testing, they were managed separately from clinical data to prevent disclosure of grouping information. The GC-MS, fully automated chemiluminescence analyzer, and HPLC instruments were calibrated before each batch of tests, and internal standards were used to ensure testing accuracy and comparability. Research data were entered in duplicate by two individuals using a specialized clinical data management system and checked for consistency. Any discrepancies were verified against the original records. After the study concluded, the database was locked and data were backed up. Clinical observers and laboratory personnel remained blinded throughout, and the random sequence was revealed by an independent individual only after the study was completed and the database locked.

### 2.6 Statistical analysis

Software called SPSS 26.0 was used for SA. According to the Shapiro-Wilk test (SWT) for normality, continuous variables that fit a normal distribution (ND) were represented by the mean ± standard deviation, whereas those that did not were represented by the median and interquartile range. For between-group comparisons, either the Mann-Whitney U test (for non-normal distributions (NND) or an independent sample t-test (for ND) were employed.

Repeated-measures ANOVA was applied for data measured repeatedly at multiple time points to analyze the interaction effect of time × group. The χ² test was used to compare categorical variables.

If any cell had a predicted frequency below 5, Fisher’s exact test was applied. The threshold for statistical significance was established at α=0.05, and all statistical tests were two-sided.

Where multiple comparisons were involved, the α level was adjusted accordingly using the Bonferroni method (e.g., α’=0.05/k). Pearson’s method was used to evaluate the correlation between changes in SCFAs and IBS-SSS, as well as the correlation between tight junction protein levels and either abdominal pain scores or the L/M ratio.

## 3 Outcomes

### 3.1 Subject screening and basic features

The basic demographic features (age, sex, and BMI), clinical characteristics (IBS duration, IBS subtype, IBS-SSS score), SCFAs, intestinal barrier indicators, and inflammatory markers of the two groups were compared. All continuous variables were determined to follow a normal distribution (P>0.05) after the SWT, and an independent sample t-test was used for comparison. The χ² test was used to compare categorical variables.

No statistically substantial variances were found (all P>0.05), indicating balanced and consistent baseline characteristics (**Table 1**).

**Table 1:** Comparison of baseline characteristics between the two groups of subjects.

Variables (units)	Probiotic group (60)	Placebo group (60)	Test value	p value
Age (years)	39.27 ± 5.36	38.62 ± 5.59	t=0.597	0.552
Sex (male/female)	22 (36.67%) / 38 (63.33%)	19 (31.67%) / 41 (68.33%)	χ²=0.326	0.568
BMI (kg/m²)	23.74 ± 2.36	23.66 ± 2.40	t=0.176	0.86
IBS subtype (D/C/M/U)	28/14/12/6 (46.67%/23.33%/20.00%/10.00%)	25/16/13/6 (41.67%/26.67%/21.67%/10.00%)	χ²=0.424	0.935
Duration (years)	4.21 ± 1.22	4.36 ± 1.28	t=0.622	0.536
IBS-SSS (0–500)	289.37 ± 35.26	286.39 ± 33.67	t=0.428	0.670
Acetate (µmol/g)	42.63 ± 5.73	42.81 ± 5.70	t=0.152	0.879
Propionate (µmol/g)	11.38 ± 1.44	11.41 ± 1.39	t=0.113	0.910
Butyrate (µmol/g)	8.72 ± 1.06	8.98 ± 1.11	t=1.221	0.225
L/M ratio	0.037 ± 0.005	0.038 ± 0.005	t=0.546	0.586
Occludin (ng/mL)	1.68 ± 0.22	1.69 ± 0.21	t=0.226	0.822
Claudin-1 (ng/mL)	2.59 ± 0.30	2.61 ± 0.29	t=0.352	0.726
Zonulin (ng/mL)	68.47 ± 8.62	68.58 ± 8.59	t=0.068	0.946
CRP (mg/L)	3.69 ± 0.54	3.73 ± 0.56	t=0.388	0.699
IL-6 (pg/mL)	6.24 ± 0.88	6.18 ± 0.85	t=0.355	0.723
TNF-α (pg/mL)	11.42 ± 1.25	11.39 ± 1.24	t=0.114	0.909
Fecal calprotectin (µg/g)	43.47 ± 5.55	43.62 ± 5.51	t=0.138	0.890

### 3.2 IBS symptom score changes

A repeated-measures ANOVA was applied to the symptom scores (IBS-SSS) of the two groups of IBS patients, revealing a significant time × group interaction effect (F=9.314, P<0.001). At each time point, between-group comparisons were then conducted using independent sample t-tests, and the Bonferroni technique was employed to alter the significance level (adjusted α=0.01).

From the 8th week of intervention to the end of follow-up, the symptom scores of the PG were considerably < than those of the PLG (all P<0.01) (**Table 2**).

**Table 2:** Changes in IBS-SSS at each time point (Mean ± SD).

Time point	Probiotic group	Placebo group	t value	p value
W0	289.37 ± 35.26	286.39 ± 33.67	0.428	0.67
W4	251.42 ± 32.58	265.18 ± 33.14	2.079	0.041
W8	223.79 ± 31.44	245.59 ± 29.62	2.742	0.007
W12	197.25 ± 26.38	230.72 ± 28.11	3.306	0.001
W16	185.04 ± 24.07	225.34 ± 27.26	3.954	<0.001
time × group interaction effect: F=9.314, P<0.001				

### 3.3 Changes in fecal SCFA levels

**Figure 1:**
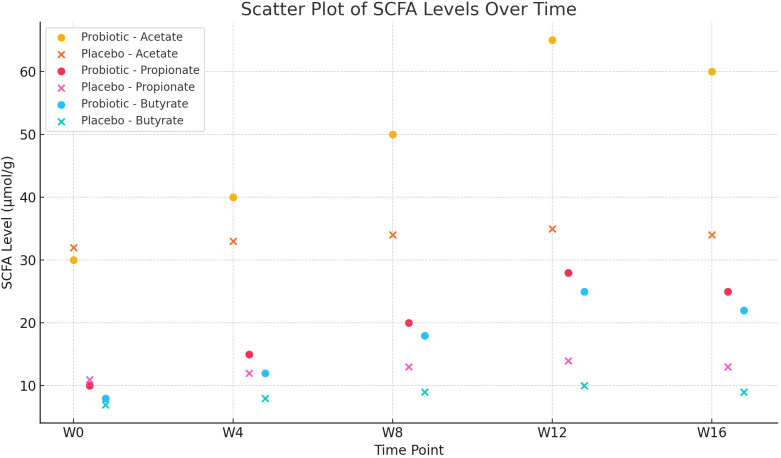
Scatter Plot of SCFA Levels Over Time.

Acetate, propionate, and butyrate concentrations in both groups’ faeces showed by Repeated-measures ANOVA, as it indicates significant group × time interaction effects (F=7.405, 6.928, and 8.263, respectively; all P<0.001). Following Week 12, the PG’s levels of acetate, propionate, and butyrate were considerably > than those of the PLG (all P<0.01), following Bonferroni adjustment of the significance threshold (α=0.01) (**Table 3**). Scatter plot showing the concentrations of acetate, propionate, and butyrate (µmol/g) in fecal samples at five time points (W0, W4, W8, W12, W16) for both probiotic and placebo groups. The plot highlights a notable increase in SCFA levels in the probiotic group over the intervention period compared to the placebo group (**Figure 1**).

**Table 3:** Comparison of SCFAs at each time point (Mean ± SD).

Time	Probiotic group	Placebo group	t Value	p Value
Acetate (µmol/g)				
W0	42.63 ± 5.73	42.81 ± 5.70	0.152	0.879
W4	45.12 ± 5.20	44.49 ± 5.08	0.603	0.551
W8	47.26 ± 5.62	44.84 ± 5.30	2.050	0.042
W12	49.63 ± 5.04	45.26 ± 5.40	3.130	0.002
W16	50.85 ± 4.89	46.09 ± 5.15	4.083	<0.001
Group × Time interaction effect: 7.405,P<0.001				
Propionate (µmol/g)				
W0	11.38 ± 1.44	11.41 ± 1.39	0.113	0.910
W4	12.15 ± 1.33	11.86 ± 1.27	1.052	0.295
W8	12.89 ± 1.49	12.12 ± 1.38	2.552	0.012
W12	13.36 ± 1.32	12.35 ± 1.33	3.712	<0.001
W16	13.58 ± 1.25	12.54 ± 1.28	4.024	<0.001
Group × Time interaction effect: F=6.928,P<0.001				
Butyrate (µmol/g)				
W0	8.72 ± 1.06	8.98 ± 1.11	1.221	0.225
W4	9.13 ± 0.95	8.85 ± 0.98	1.443	0.152
W8	9.49 ± 0.90	9.04 ± 0.92	2.474	0.015
W12	9.90 ± 0.89	9.18 ± 0.93	4.028	<0.001
W16	10.05 ± 0.82	9.21 ± 0.85	5.154	<0.001
Group × Time interaction effect: F=8.263, P<0.001				

### 3.4 Intestinal barrier function

For intestinal permeability (L/M ratio) and tight junction proteins (Occludin, Claudin-1, Zonulin) in the two subject groups, a significant group × time interaction effect (TIE) was found by the repeated measures ANOVA (F values were 8.956, 9.347, 4.118, and 5.654, respectively; all P<0.05).

After applying the Bonferroni method to correct the significance level (adjusted α=0.0167), the intestinal permeability (L/M ratio) and Occludin concentration were both significantly better than those of the PLG at Week 8 and Week 12 (both P<0.0167). Claudin-1 and Zonulin concentrations were only significantly better at Week 12 compared to the PLG (both P<0.0167) (**Table 4**).

**Table 4:** Changes in intestinal permeability (L/M ratio) and tight junction proteins at each time point (Mean ± SD).

Time	Probiotic group	Placebo group	t value	p value
Intestinal Permeability (L/M Ratio)				
W0	0.037 ± 0.005	0.038 ± 0.005	0.546	0.586
W8	0.032 ± 0.004	0.036 ± 0.004	4.228	<0.001
W12	0.029 ± 0.003	0.035 ± 0.005	6.105	<0.001
Group × Time interaction effect: F=8.956, P<0.001				
Occludin (ng/mL)				
W0	1.68 ± 0.22	1.69 ± 0.21	0.226	0.822
W8	1.87 ± 0.24	1.72 ± 0.20	2.631	0.01
W12	2.01 ± 0.25	1.77 ± 0.21	4.826	<0.001
Group × Time interaction effect: F=9.347, P<0.001				
Claudin-1 (ng/mL)				
W0	2.59 ± 0.30	2.61 ± 0.29	0.352	0.726
W8	2.73 ± 0.28	2.63 ± 0.27	1.883	0.063
W12	2.84 ± 0.27	2.66 ± 0.28	3.047	0.003
Group × Time interaction effect: F=4.118, P=0.019				
Zonulin (ng/mL)				
W0	68.47 ± 8.62	68.58 ± 8.59	0.068	0.946
W8	64.12 ± 7.56	67.29 ± 7.82	2.142	0.035
W12	61.26 ± 7.24	66.83 ± 7.76	3.942	<0.001
Group × Time interaction effect: F=5.654, P=0.005				

### 3.5 Inflammatory and immune indicators

For the inflammatory markers TNF-α, IL-6, CRP, and calprotectin, repeated measures ANOVA revealed significant group × TIE in both groups (F values of 7.832, 6.439, 4.571, and 5.812, respectively; all P<0.05).

After Bonferroni correction of the significance level (adjusted α=0.0167), these indicators were considerably lesser in the PG than in the PLG only at the 12th week of intervention (all P<0.0167) (**Table 5**).

**Table 5:** Changes in CRP, IL-6, TNF-α, and Calprotectin at each time point (Mean ± SD).

Time	Probiotic group	Placebo group	t value	p value
CRP (mg/L)				
W0	3.69 ± 0.54	3.73 ± 0.56	0.388	0.699
W8	3.28 ± 0.48	3.49 ± 0.50	1.990	0.049
W12	2.95 ± 0.44	3.44 ± 0.51	4.522	<0.001
Group × Time interaction effect: F=7.832,P=0.001				
IL-6 (pg/mL)				
W0	6.24 ± 0.88	6.18 ± 0.85	0.355	0.723
W8	5.72 ± 0.81	6.07 ± 0.79	2.168	0.032
W12	5.31 ± 0.73	5.97 ± 0.80	4.245	<0.001
Group × Time interaction effect: F=6.439,P=0.002				
TNF-α (pg/mL)				
W0	11.42 ± 1.25	11.39 ± 1.24	0.114	0.909
W8	10.86 ± 1.11	11.21 ± 1.19	1.596	0.114
W12	10.34 ± 1.02	11.06 ± 1.18	3.497	0.001
Group × Time interaction effect: F=4.571,P=0.012				
Calprotectin (µg/g)				
W0	43.47 ± 5.55	43.62 ± 5.51	0.138	0.890
W8	39.83 ± 5.14	41.82 ± 5.22	1.940	0.055
W12	36.94 ± 5.07	40.82 ± 5.20	3.893	<0.001
Group × Time interaction effect: F=5.812,P=0.004				

### 3.6 Adverse events and compliance

There were no statistically significant changes in the occurrence of adverse events, including rash, nausea, dizziness, and increased pain in the abdomen, between the two groups during the intervention (χ² test, all P>0.05). Furthermore, there was no statistically major variance in compliance among the 2 groups according to the χ² test (P>0.05) (**Table 6**).

**Table 6:** Comparison of adverse events and compliance during the intervention (n, %).

Item	Probiotic group	Placebo group	χ² value	p value
Aggravated abdominal pain	7 (11.67%)	10 (16.67%)	0.598	0.439
Rash	2 (3.33%)	5 (8.33%)	1.403	0.237
Dizziness	3 (5.00%)	2 (3.33%)	0.209	0.647
Nausea	4 (6.67%)	6 (10.00%)	0.451	0.502
Compliance ≥80%	56 (93.33%)	54 (90.00%)	0.379	0.538

### 3.7 Correlation among SCFAs and changes in IBS-SSS

**Figure 2:**
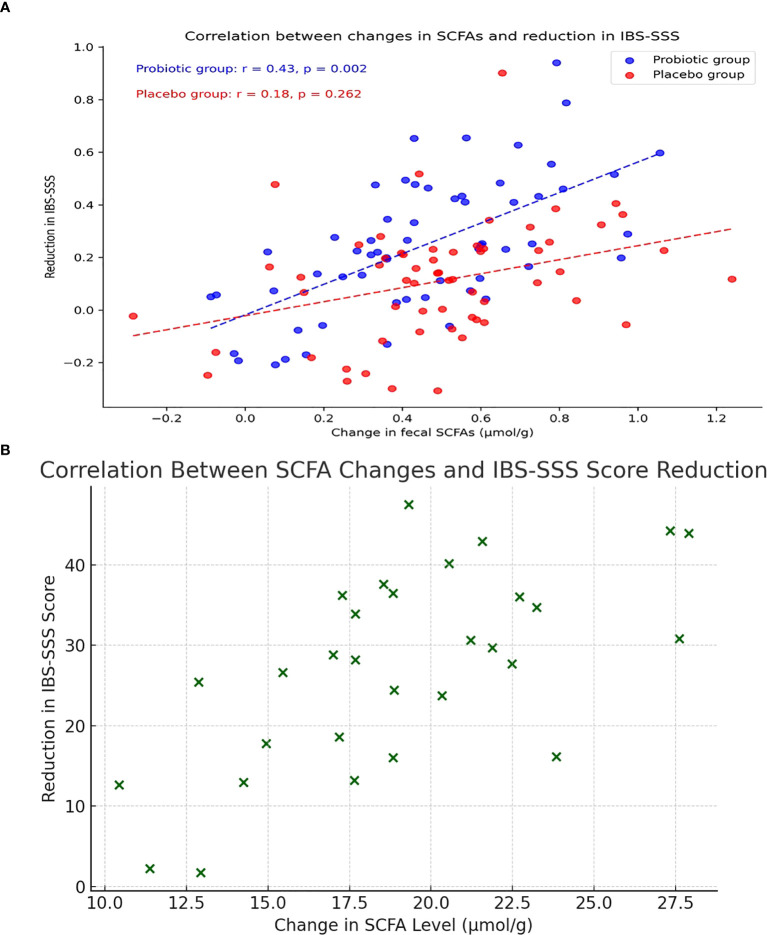
Correlation Between SCFA changes and IBSS-SSS score.

According to Pearson correlation analysis, the PG’s rise in SCFAs and fall in IBS-SSS scores from baseline to Week 12 shown a strong positive correlation (r=0.43, P=0.002), whereas the PLG exhibited no significant correlation (r=0.18, P=0.262) (**Figure 2A**). Scatter plot illustrating the correlation between changes in total SCFA levels (µmol/g) and reductions in IBS Symptom Severity Score (IBS-SSS) from baseline to Week 12 in the probiotic group (**Figure 2B**).

### 3.8 Subgroup analysis of the PEP

The subgroup analyses based on different IBS subtypes (PEP: IBS-SSS), the PG’s IBS-D, IBS-C, IBS-M, and IBS-U subtypes all exhibited significantly lower symptom scores at Week 12 compared to the PLG (all P<0.05) using the t-test or Mann-Whitney U test (**Table 7**).

**Table 7:** Subgroup analysis by IBS subtype or age (Mean ± SD).

IBS-SSS score	Probiotic group	Placebo group	Test value	p value
IBS-D (n=28/25)				
W0	291.65 ± 32.14	289.38 ± 33.08	t=0.279	0.783
W12	197.42 ± 26.25	228.75 ± 27.36	t=4.271	<0.001
IBS-C (n=14/16)				
W0	296.48 ± 34.12	294.37 ± 32.57	t=0.170	0.866
W12	205.27 ± 22.18	246.53 ± 25.84	t=4.868	<0.001
IBS-M (n=12/13)				
W0	282.39 ± 35.06	280.76 ± 32.74	t=0.132	0.896
W12	193.48 ± 28.13	213.62 ± 29.74	t=2.047	0.048
IBS-U (n=6/6)				
W0	296.52 [284.66, 307.33]	294.58 [287.31, 305.27]	U=13.000	0.778
W12	202.49 [191.26, 210.57]	245.36 [234.22, 252.59]	U=1.000	0.004

## 4 Discussion

The improvement in symptoms of IBS patients through probiotics was fully demonstrated in this study. After the intervention began, the overall symptom scores in the probiotic group showed a continuous downward trend. The difference from the control group became apparent starting from the eighth week and reached a more significant level at the twelfth week. This trend of improvement was seen in a variety of IBS subtypes, including mixed, constipation-predominant, diarrhea-predominant, and unclassified. This suggests that the effects of probiotic intervention are not specific to any one subtype but rather have a wider range of applications (Umeano et al., 2024; de Chambrun et al., 2015).

A decrease in symptom scores not only indicates relief from discomfort such as AP and bloating but also reflects an enhanced subjective perception of bowel habits and quality of life. Probiotics may regulate the balance of the intestinal microecology, helping reduce excessive proliferation of harmful bacteria while promoting the colonization of protective strains. This in turn inhibits the production of inflammatory factors in the intestine, alleviates local inflammation, and lowers visceral sensitivity (Goodoory et al., 2023; Gareau et al., 2010). Correspondingly, this study uniformly stipulated basic measures for all subjects in terms of diet, exercise, and psychological interventions to maintain a relatively stable external environment, thereby allowing a fuller demonstration of the effects of probiotics in reducing symptoms and optimizing intestinal function. Compared with sole pharmacological intervention, this comprehensive management strategy more closely simulates real clinical settings and enhances the generalizability of the conclusions. Numerous earlier research investigations on the beneficial impacts of probiotics in the diagnosis of IBS have concentrated on either single-subtype findings or short-term interventions (Ghuloom et al., 2023; Hod et al., 2017). Through longer-term and more comprehensive follow-up evaluations, this study further confirms that probiotics benefit patients across multiple subtypes, with more stable effects seen after more than eight weeks of intervention. The results show that when probiotics fully exert their effect within a relatively suitable timeframe, clinical symptoms may continue to improve. Meanwhile, the three core symptoms—abdominal pain, diarrhea, and constipation—all showed some degree of improvement, indicating a potential synergistic effect of probiotics through multiple pathways such as mucosal barrier repair, regulation of intestinal smooth muscle function, and reduced visceral sensory sensitivity. In practical applications, attention should be paid to the coordination of probiotics with lifestyle interventions to achieve better symptom control and quality of life improvements.

In this study, SCFA was closely related to a reduction in the symptoms of IBS.The test outcomes indicate that from the twelfth week of intervention onward, levels of acetate, propionate, and butyrate in the PG significantly increased, while patients’ subjective symptom scores continued to decline, suggesting that after a certain period of probiotic intake, the intestinal microecological environment underwent beneficial changes (Tuteja et al., 2022; El-Salhy et al., 2021). SCFAs are crucial for immunological regulating and inflammatory responses in addition to providing intestinal epithelial cells (IEC) with energy.

This process may effectively mitigate damage to the intestinal mucosa and reduce stimulation caused by increased epithelial permeability (Zhang et al., 2022; Parada Vengas et al., 2019). Butyrate, widely recognized as a protective factor for the intestinal mucosa, is crucial for maintaining the integrity of the mucosal barrier. It may enhance the expression and function of TJP, thereby preventing exogenous irritants or pathogens from entering the intestinal wall layer (Abdel-Samie et al., 2021; Peng et al., 2009). In this research, the variance in SCFA stages among the 2 groups became evident after a longer duration, indicating that probiotics must colonize the intestine and continuously ferment available substrates in order to produce these metabolites to a greater extent later on, resulting in sustained and more pronounced symptom relief. Compared to previous studies conducted mostly *in vitro* or in animal models, this trial provides clinical evidence showing that elevated SCFAs are closely associated with decreased subjective symptom scores and can form a synergistic effect with the restoration of intestinal barrier function. The correlation analysis (r=0.43, P=0.002) further supports a potential causal chain of “probiotics—SCFAs—intestinal barrier—symptom relief,” and suggests that probiotic combinations may serve as an important strategy in clinical practice for addressing increased intestinal mucosal permeability (Asha and Khalil, 2020; Garcia Mansilla et al., 2024). Throughout the intervention, all subjects adhered to a unified basic intervention protocol, significantly reducing external confounding factors and highlighting the core regulatory role that SCFAs may play in improving the intestinal environment and protecting the epithelial barrier. Clinically, how to select suitable probiotic preparations and dietary fiber combinations for different populations or stages of disease progression may require deeper exploration and stratified management in future studies, so that more IBS patients of different subtypes can benefit and establish a more precise and practicable treatment model.

Significant improvements in the intestinal barrier and simultaneous reductions in inflammatory indicators observed in this study provided robust support for alleviating irritable bowel syndrome symptoms. The decrease in the L/M ratio and the increase in tight junction proteins (Occludin, Claudin-1, Zonulin) indicate that mucosal integrity was maintained, inhibiting harmful intraluminal factors from invading the intestinal wall. The marked decrease in inflammatory indicators such as CRP, IL-6, TNF-α, and calprotectin during the later stages of the intervention further suggests a gradual easing of immune activation triggered by barrier dysfunction. This positive cycle may depend on the sustained production of metabolites like SCFA, which provide energy for IEC and help maintain microecological balance, thereby reducing mucosal inflammation and enhancing resistance to external stimuli (Wang et al., 2019). Between Weeks 8 and 12, the probiotic group not only demonstrated declining clinical symptom scores but also exhibited restored intestinal barrier function and improved inflammatory status, affirming the deep interconnectedness among “barrier stability—inflammation relief—symptom improvement” (Connell et al., 2018). In this trial, monitoring permeability and multiple inflammatory markers over a relatively long follow-up period offered a more comprehensive empirical basis for exploring the interaction between mucosal repair and immune intervention. Compared with previous studies that focused solely on changes in intestinal permeability or immune factors, this holistic observation more clearly highlights the synergistic integrative effects of probiotics on both physiological and pathological processes. Past IBS interventions that overlooked dual regulation of the intestinal barrier and inflammatory burden often yielded only short-term or partial symptom relief (Wei et al., 2025). The outcomes of this research suggest that an integrated approach targeting the mucosal barrier and inflammatory factors can achieve a deeper level of improvement in intestinal homeostasis, providing more enduring and multidimensional benefits for IBS patients. Notably, in this study, all subjects received standardized dietary and exercise management, which further minimized external interference and maximized the potential of probiotics to restore the mucosa and inhibit inflammation. Future work could attempt to clarify the specific mechanisms involved while refining probiotic combinations and dosages, thereby offering more insight for personalized IBS treatment in clinical practice and laying the groundwork for managing other related intestinal functional disorders.

## 5 Limitations

Subjects for this study were largely concentrated in one area, and it was carried out in a single centre. Due to differences in lifestyle and microecology among populations in different regions, further multi-center research across a broader scope is needed to verify the generalizability of its conclusions. The intervention period was set at twelve weeks, which, although it can preliminarily reveal the effectiveness of probiotics in alleviating symptoms, repairing the mucosal barrier, and modulating inflammation, does not clarify long-term effects. The follow-up period should be prolonged in future research to ascertain how long probiotic therapies last.

This study only carried out conventional microecological assessments and clinical indicator evaluations without incorporating microbiome and metabolome analyses, making it impossible to deeply characterize changes in probiotic structure and metabolic pathways at the microbiome level. In future research on a larger scale and across multiple centers, it is recommended to integrate high-throughput sequencing and metabolomics technologies to more precisely explore changes in microbiome structure, functional genes, and metabolites, thereby further elucidating the mechanisms and pathways of probiotic action. This would allow for tailored probiotic combinations and personalized dietary plans for different IBS subtypes. The selection of probiotic strains and related excipient formulations may be further optimised by combining multi-omics data with artificial intelligence analysis, creating a more focused and useful customised therapy model.

The unified basic intervention model used in this study could also be optimized to incorporate differentiated exercise and psychological adjustment programs according to patients’ varying stages of the disease, in order to evaluate their synergistic effects with probiotic interventions.

## 6 Conclusion

This study demonstrates that probiotics, under a unified basic intervention, can significantly relieve AP, bloating, and abnormal defecation in patients with IBS, with symptom scores showing a marked difference from the control group beginning in the eighth week and continuous improvement across multiple subtypes by the twelfth week. There is a strong correlation between elevated short-chain fatty acid levels and clinical symptoms, while stabilization of the intestinal barrier and modulation of the inflammatory state further reinforce the overall efficacy. The restoration of intestinal permeability and tight junction proteins, as well as the reduction in inflammatory markers such as CRP, IL-6, TNF-α, and Calprotectin, provide reliable evidence for the role of probiotics in mucosal protection and immune regulation. The results suggest that a multi-layered, coordinated intervention targeting the mucosal barrier, inflammatory burden, and microecological balance can offer more enduring and extensive clinical benefits for irritable bowel syndrome.

## Data Availability

The raw data supporting the conclusions of this article will be made available by the authors, without undue reservation.
